# A multidisciplinary approach to managing rib fracture non- or malunion with bridging heterotopic ossification: a case report and surgical technique

**DOI:** 10.1093/jscr/rjag746

**Published:** 2026-08-26

**Authors:** Ahmed Faidh Ramzee, Ahmad AlJabary, Ruben Peralta, Murad Alahmad, Mushreq Al-Ani, Hamdan Hamdan, Mohammed Haji, Nitasha Saleem, Sandro Rizoli, Ayman El-Menyar, Hassan Al-Thani, Talat Chughtai

**Affiliations:** Department of Trauma Surgery, Hamad Medical Corporation, PO Box 3050, Doha, Qatar; Department of Anesthesia and Chronic Pain, Hamad Medical Corporation, PO Box 3050, Doha, Qatar; Department of Trauma Surgery, Hamad Medical Corporation, PO Box 3050, Doha, Qatar; Department of Surgery, Universidad Nacional Pedro Henriquez Urena, Santo Domingo 10100, Dominican Republic; Department of Trauma Surgery, Hamad Medical Corporation, PO Box 3050, Doha, Qatar; Department of Trauma Surgery, Hamad Medical Corporation, PO Box 3050, Doha, Qatar; Department of Anesthesia and Chronic Pain, Hamad Medical Corporation, PO Box 3050, Doha, Qatar; Department of Anesthesia and Chronic Pain, Hamad Medical Corporation, PO Box 3050, Doha, Qatar; Department of Trauma Surgery, Hamad Medical Corporation, PO Box 3050, Doha, Qatar; Department of Trauma Surgery, Hamad Medical Corporation, PO Box 3050, Doha, Qatar; Department of Trauma Surgery, Hamad Medical Corporation, PO Box 3050, Doha, Qatar; Department of Clinical Medicine, Weill Cornell Medicine, PO Box 24144, Doha, Qatar; Department of Trauma Surgery, Hamad Medical Corporation, PO Box 3050, Doha, Qatar; Department of Trauma Surgery, Hamad Medical Corporation, PO Box 3050, Doha, Qatar

**Keywords:** rib fractures, chest wall injury, malunion, SSRF radiofrequency ablation, pain management

## Abstract

Rib nonunion with malunion and heterotopic ossification (HO) is a rare complication of rib fractures. It is associated with significant morbidity and quality-of-life impairments owing to pain. Management is complex and requires a multidisciplinary approach involving surgery and appropriate multimodal pain management strategies, including systemic and locoregional interventions. We report a case of rib fracture nonunion with malunion and HO formation resulting in significant disability, successfully managed with surgical intervention and chronic pain management strategies employing radiofrequency ablation.

## Background

Nonunion rib fracture occurs when there is no osseous bone healing within 9 months or no evidence of radiographic healing after 3 months of injury [1]. Malunion is defined as an improperly healed fracture causing displacement and angulation of the rib. Heterotopic ossification (HO) is the abnormal growth of lamellar bone replacing soft tissue [2]. Almost 5%–10% of patients with rib fractures are reported to develop chronic nonunion [2, 3]. These complications can lead to significant morbidity owing to chronic pain from possibly entrapped intercostal nerves.

## Case report

**Figure 1:**
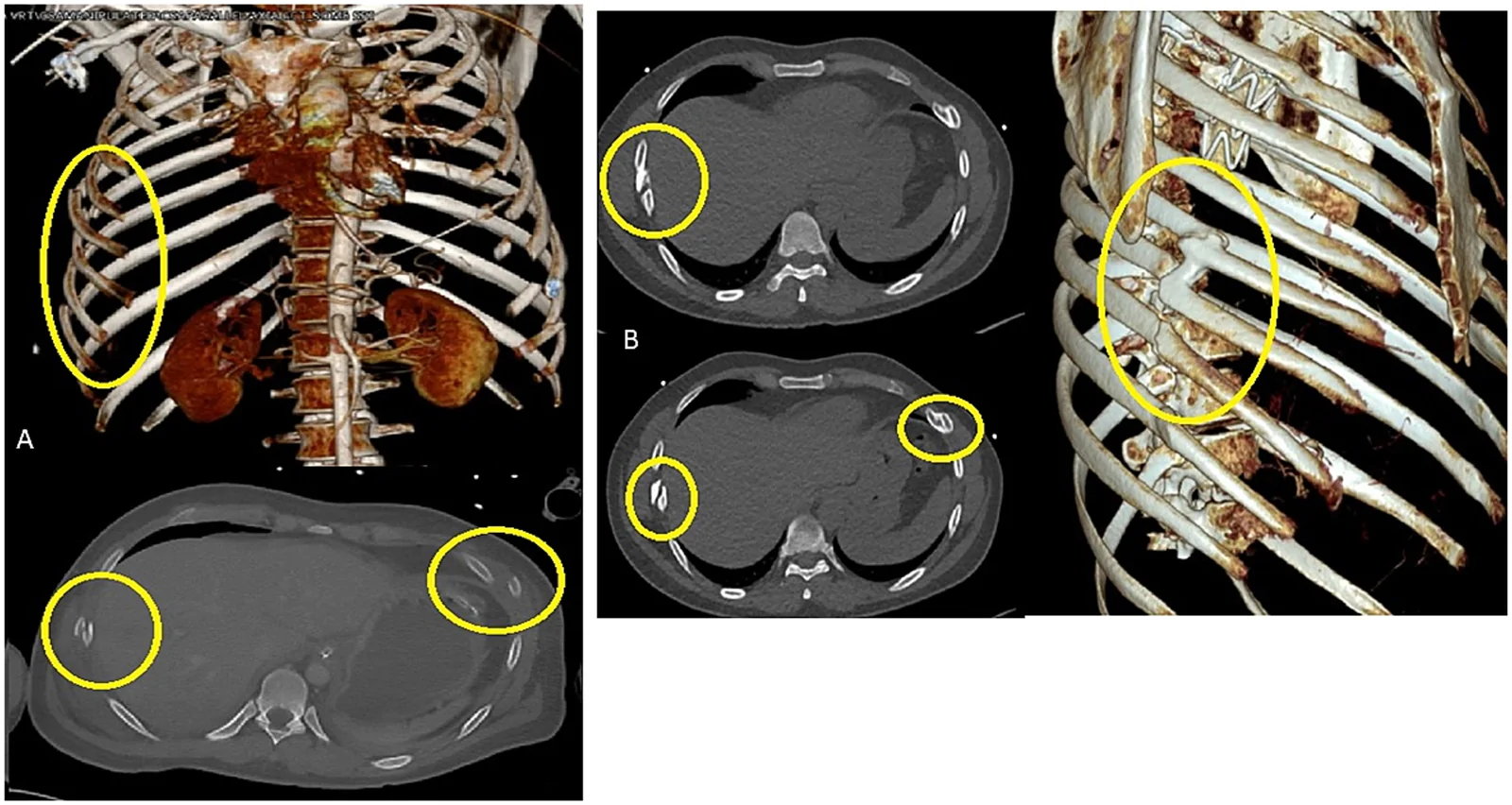
(A) Initial CT with 3D reconstruction showing bilateral fractures; (B) CT with 3D reconstruction showing malunion of the sixth and seventh rib with heterotopic ossification and nonunion of the eighth rib.

A 37-year-old male was admitted following a motor vehicle collision. He was hypotensive and was intubated. Investigations revealed a Type B aortic dissection, solid organ injury, bilateral rib fractures with hemothorax (right-sided ribs 1, 3–10 and left-sided ribs 1, 2–9). The left-sided fractures were more significantly displaced than the right (Fig. 1). He was not a current smoker and was previously healthy. The patient underwent endovascular aortic repair, and femoral intramedullary nailing. The abdominal injuries and rib fractures were managed conservatively (multimodal systemic analgesia). He was discharged on Day 15 with a regular follow-up.

**Figure 2:**
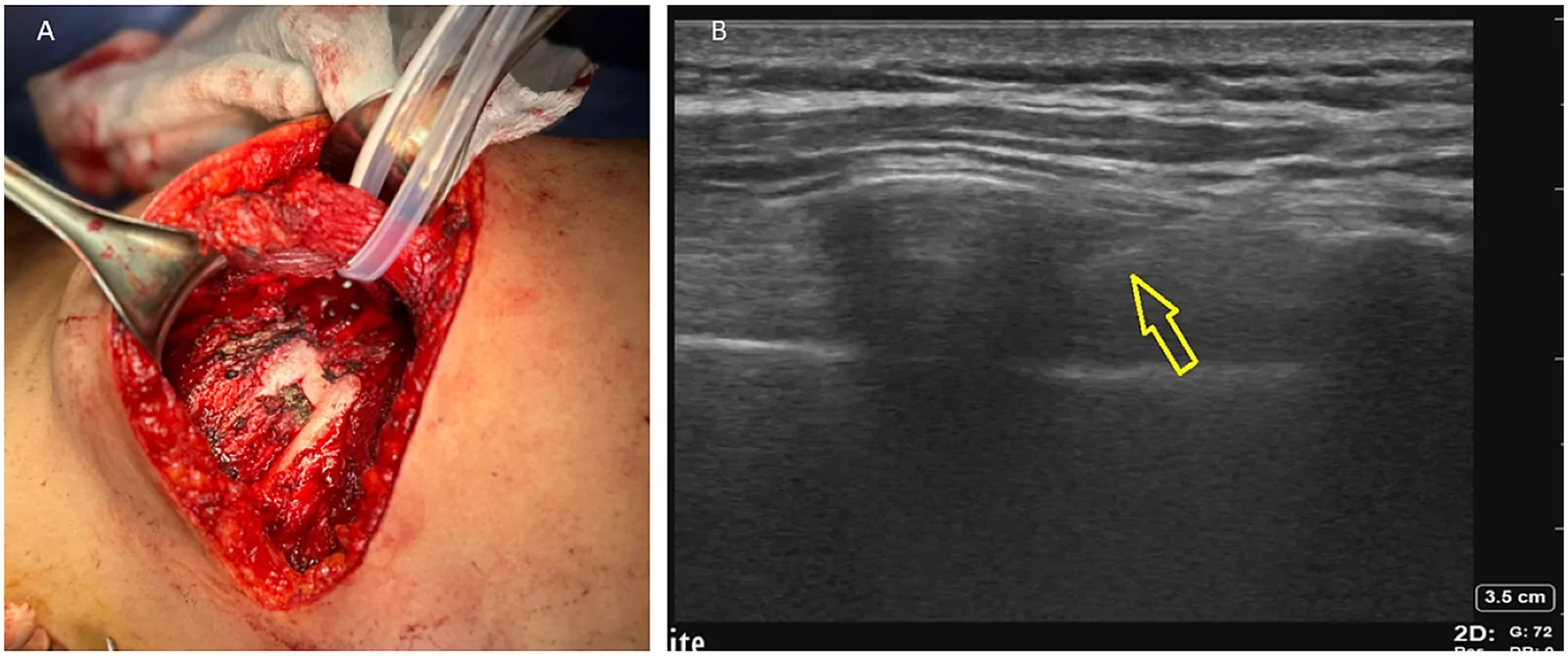
(A) Intraoperative photo showing the heterotopic ossification between ribs 6 and 7; (B) ultrasound-guided RFA with needle tip positioned between the innermost and internal intercostal muscles.

A year later, he presented with debilitating right-sided chest pain, reporting a clicking sensation on the right lateral chest wall without a history of recent trauma. He reported a pain score of 8–9 out of 10 constantly (numeric rating scale, NRS). He was unable to ambulate due to the pain and was unable to work for the last 2 months. Repeat computed tomography (CT) imaging confirmed a fractured segment of the right-sided seventh rib, malunited with the sixth rib above, with formation of a bridging HO, and there was nonunion of the eighth rib (Fig. 1). The left-sided rib fractures with significant displacement had healed appropriately. The patient was counseled that the malunion and nonunion were probably causing nerve impingement, leading to his symptoms. He was given the option of surgical fixation to possibly alleviate his pain. The surgery was done in the left lateral decubitus position via a lateral incision over the seventh rib. A muscle-sparing approach was employed. A large callus was noted between the sixth rib and the anterior segment of the seventh rib with nonunion of the seventh rib. The eighth rib was nonunited (Fig. 2). The callus was resected. The seventh intercostal nerve was identified and inadvertently cut; the ends were tied off. Both the seventh and eighth ribs were fixed with the MatrixRIB™ Fixation System (DePuy Synthes – Johnson & Johnson) plates and screws. A serratus anterior catheter was placed for postoperative pain management. The postoperative course was uneventful, and pain was well controlled. He was discharged on Day 5 with a significantly decreased pain score of 3–4 out of 10. Two weeks later, he exhibited a full range of motion at the shoulder joint. After 45 days, he was able to resume his daily activities.

**Figure 3:**
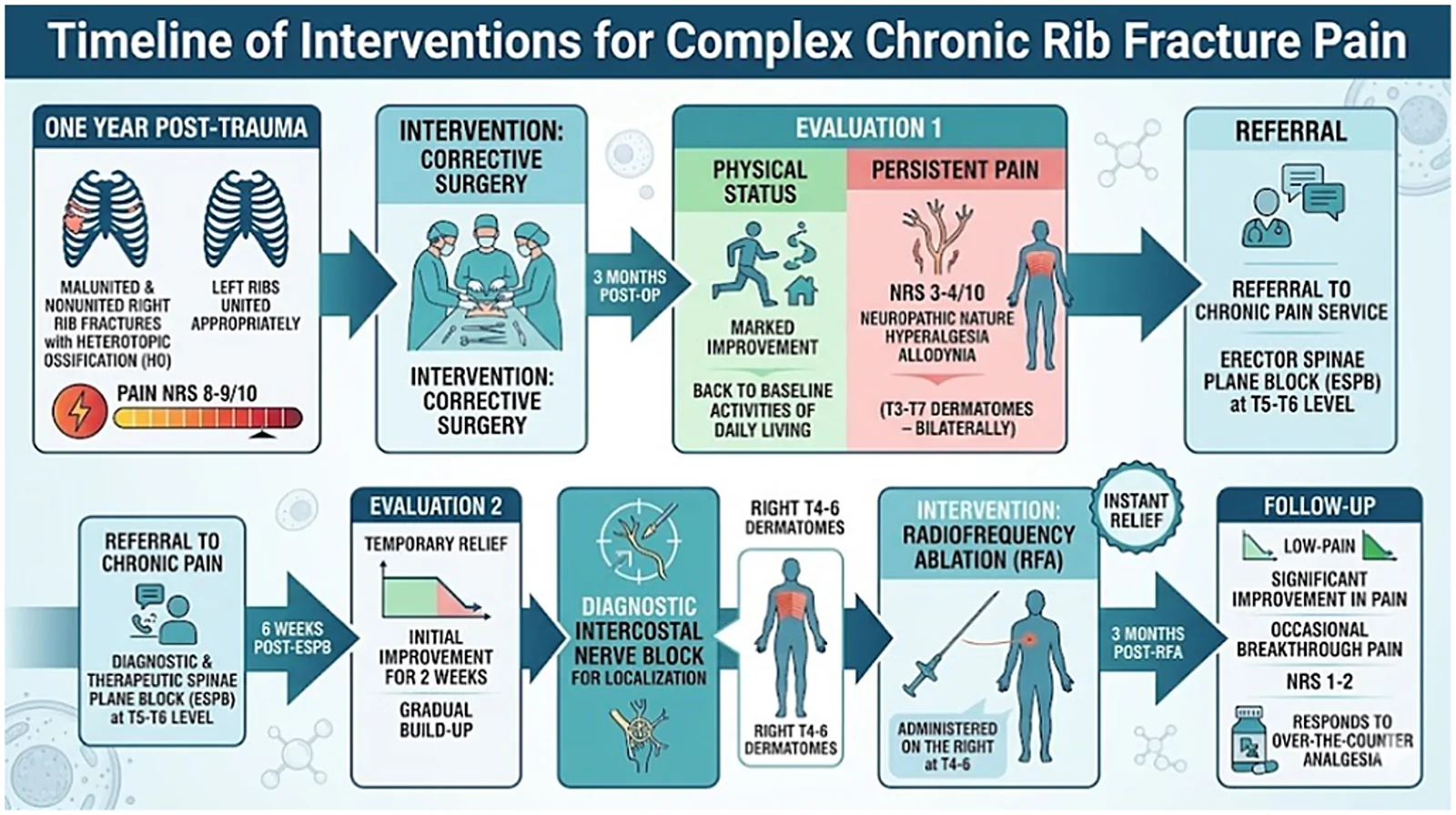
Timeline of interventions for complex chronic rib fracture pain.

The pain was incompletely alleviated, although significantly improved. He had neuropathic pain with hyperalgesia and allodynia. He was on paracetamol, celecoxib, and amitriptyline at this time. A session of erector spinae block with 0.25% levobupivacaine followed by a diagnostic intercostal nerve block 6 weeks later was administered, and the problematic dermatomes were identified at T4–6 on the right. Thermal radiofrequency ablation (RFA) with the Cosman G4 Radiofrequency Generator (Boston Scientific, Marlborough, MA) was performed. A monopolar 10 mm needle tip probe was positioned between the innermost and internal intercostal muscles, activated to 80°C for 90 s (Fig. 2). Instant pain relief was noted (1–2 on NRS). At 8 months (3 months post RFA), he reports complete pain relief with occasional breakthrough pain, which responds to over-the-counter analgesics. He is back to his baseline in daily living functions and has returned to work with no untoward complications from either the surgical or RFA interventions. Figure 3 describes the timeline of interventions for complex chronic rib fracture pain.

## Discussion

This case highlights a complex outcome of aberrant rib fracture healing and underscores the need for a multidisciplinary approach, including surgical correction and appropriate long-term pain management interventions such as RFA. Most nonunion/malunions occur in the lower ribs in the lateral and posterolateral positions [4]. Increased mobility of the lower chest wall as compared to the upper chest wall, the attachment of abdominal wall musculature to the lateral lower chest wall, and the attachment of the diaphragm to the lower ribs result in multidirectional, rotational forces on the lower ribs, potentially preventing adequate rib apposition and inadequate healing [5].

The Chest Wall Injury Society recommendations for surgical stabilization of nonunited rib fractures advise referral of patients with persistent pain at 3–6 months post-injury with radiographic evidence of nonunion to a chest wall specialist [3]. Management of these pathologies poses a challenge and requires a multidisciplinary approach, including multimodal pain management, regional anesthesia, and surgical stabilization [3]. No randomized controlled studies exist comparing surgical intervention with other nonoperative options. Existing evidence, although of low quality, shows that surgery may improve pain, reduce opiate requirements, and improve patient-reported outcomes [3]. In a prospective study of 15 patients undergoing surgery for rib fracture nonunion, a significant improvement in quality of life indices was noted [5].

Although pain was significantly improved, it was not completely resolved in most reported cases. As much as 20%–35% of patients may have chronic pain post rib fracture, and the incidence of malunion and nonunion is uncertain [6]. Apart from long-term opioid use for chronic pain, intercostal blocks and cryoablation are viable options; however, their effects are short-lasting [7, 8]. RFA has been described in the management of pain from spinal pathologies; however, its use for intercostal neuralgia associated with rib fractures is less frequently reported [7, 8]. A small series of patients who underwent RFA had good outcomes. The effects of RFA may last 6 months with minimal complications: bleeding, infection, and pneumothorax in the acute setting and neuroma formation in the long term [8]. As a minimally invasive procedure that can be performed on an outpatient basis, its potential value in managing chronic intercostal neuralgia post-rib fractures should be considered, and larger-scale studies are recommended to assess its efficacy.

## Conclusion

Rib fractures with malunion and nonunion, and HO, represent significant challenges for both patients and physicians. While surgical correction will be required in most cases, neuralgic pain requires a more nuanced approach and long-term options such as RFA.

## Data Availability

No new data were created or analyzed in this study.
